# A Randomized Controlled Pilot Trial to Test the Efficacy of Intranasal Chlorpheniramine Maleate With Xylitol for the Treatment of Allergic Rhinitis

**DOI:** 10.7759/cureus.14206

**Published:** 2021-03-31

**Authors:** Marcos Sanchez-Gonzalez, Syed A Rizvi, Joselit Torres, Gustavo Ferrer

**Affiliations:** 1 Graduate Medical Education, Lake Erie College of Osteopathic Medicine, Erie, USA; 2 Graduate Medical Education, Larkin Community Hospital, South Miami, USA; 3 Asthma, Allergy and Immunology, Vargas Hospital Caracas, Caracas, VEN; 4 Pulmonary Critical Care, Aventura Hospital and Medical Center, Aventura, USA

**Keywords:** rhinitis, allergy, chlorpheniramine maleate, xylitol

## Abstract

The prevalence of allergic rhinitis (AR), including symptoms of sneezing, nasal itching, airflow obstruction, and nasal discharge caused by histamine and immunoglobulin E (IgE)-mediated reactions, is ~30% in the U.S. Recent studies seem to suggest that the allergic inflammatory processes in AR may be induced by the interaction between an allergen (trigger) and the nasal microbiome (substrate). In this study, we have identified two agents with antihistaminic and microbiome-modulating characteristics that can be administered intranasally, namely, chlorpheniramine maleate (CPM) and xylitol (X). This study aimed to test the efficacy of intranasal CPM plus xylitol (CPM+X) nasal for the treatment of AR in an outpatient setting. A multicenter, randomized, double-blind, 30-day pilot study was conducted during the spring of 2019. After starting five days of placebo therapy (run-in period), patients with moderate-to-severe AR nasal symptoms were randomized to treatment with CPM+X (n=16) spray and nasal saline placebo (PLB; n=13). Both treatments were administered in the form of one spray dose (~100 µL of the solution containing 1.25 mg CPM) per nostril twice a day. Outcome variables were the changes in visual analog scale (VAS) and daily symptoms score (DSS) at days 1, 5, 10, 15, 25, and 30 after the initiation of the treatment. ANOVA (analysis of variance) with repeated revealed a significant treatment-by-time interaction such that the CPM+X group had a significant decrease (p < 0.05) in both DSS (∆-3.0 ± 2.7) and VAS (∆-3.8 ± 2.0) scores compared to PLB after 30 days. The difference in DSS and VAS scores between the groups was evident just after five days (day 10) of using CPM+X. The CPM+X scores were significantly lower (p < 0.008) starting from day 10 compared with day 1, whereas there were no statistically significant (p > 0.008) changes in the PLB during the 30-day treatment window. The present data suggest that nasal CPM+X use effectively improves AR symptoms. A large-scale study of the long-term effects of CPM+X for the treatment of other chronic respiratory disorders and the potential microbiome-modulating effects warrants further investigation.

## Introduction

The prevalence of allergic rhinitis (AR), including symptoms of sneezing, nasal itching, airflow obstruction, and nasal discharge caused by histamine and immunoglobulin E (IgE)-mediated reactions, is ~30% in the U.S. [1]. In addition to negatively affecting the quality of life and productivity, the presence of AR, both seasonal and perennial, significantly increases the likelihood of more serious chronic respiratory conditions and disorders [2]. Since the nose represents more than 50% of the total resistance of the upper airway, playing an important role in the establishment of physiological functions such as humidification, heating, and air filtration, the presence of AR affects the nasal cycles and thus increase the risk of asthma and sleep apnea [3].

The main underlying pathophysiological processes accountable for AR are not completely understood. However, recent studies seem to suggest that the allergic inflammatory processes in AR may be induced by the interaction between an allergic agent or allergen (trigger) and the nasal microbiome (substrate) [2,4]. For the aforementioned reasons, intranasal therapies capable of decreasing the allergic inflammatory responses with the potential to positively modulate the microbiome should be prioritized to find better and more effective treatments against AR [2].

In this study, we identified two candidate agents with antihistaminic and microbiome-modulating characteristics that can be administered intranasally, namely, chlorpheniramine maleate (CPM) and xylitol. Available for many years, oral CPM is considered a low-cost and safe first-generation antihistamine displaying antiallergic, anti-inflammatory, bronchodilatory, and antiviral properties [5-7]. Nevertheless, the intranasal use of CPM has been poorly explored. Interestingly, it has been documented that aerosolized and intranasal CPM is an effective means to manage exercise-induced asthma and is as effective as oral dose to increase systemic levels of the drug [8,9].

A sweetener with antimicrobial and anti-inflammatory properties, xylitol, has been shown effective in improving chronic rhinitis as well displaying as important microbiota and immunological modulatory effects [10,11]. It is worth noting that as a candidate for the treatment of AR, xylitol may be applied intranasally as well as display excellent mucolytic effects [10,12]. It is our rationale that the combination of therapeutic agents delivered intranasally and capable of reducing the main components of the allergic and inflammatory cascade as well as providing microbiome modulation effects would provide a dual almost synergistic beneficial effect for the effective treatment of AR. Accordingly, the main objective of this study was to test the efficacy of intranasal CPM plus xylitol (CPM+X) for the treatment of AR in an outpatient setting. It was hypothesized that CPM+X would be effective in decreasing the symptoms associated with AR in an outpatient setting.

## Materials and methods

Patients

A total of 29 patients (F= 8) gave written informed consent, as approved by the Institutional Ethics Committee as part of the study. The inclusion criteria were met the study inclusion of diagnosis of AR, 18-65 years of age, and not using oral steroids, oral antihistamines, or naphazoline during the three months before enrollment. Patients with surgical treatment for nasal polyps during the last three months, cystic fibrosis, purulent nasal infection, and any disease likely to interfere with the study parameters or which gave evidence of any serious or unstable concurrent disease or psychological disorder were excluded from participating in the study.

Study design

A multicenter, randomized, double-blind, 30-day pilot study was conducted during the spring of 2019. Randomization and matching were performed by someone not associated with the care or assessment of the patients by means of a computer-generated random number table (with a 20% random element) using an allocation ratio of 1:1 [13]. After starting five days of placebo therapy (run-in period), patients with moderate-to-severe AR nasal symptoms were randomized to treatment with CPM+X (n=16) spray and nasal saline placebo (PLB; n=13). Both treatments were administered in the form of one spray dose (~100 µL of the solution containing 1.25 mg CPM) per nostril twice a day.

Outcomes and scales

Outcome variables were the changes in visual analog scale (VAS) and daily symptoms score (DSS) at day 1, 5, 10, 15, 25, and 30 after the initiation of the treatment. Briefly, the symptom scores were measured using a four-point severity rating scale ranging from 0 to 3 (0 = no symptoms; 1 = mild symptoms; 2 = moderate symptoms; and 3 = severe symptoms). The DSS consists of six individual symptom scores: four nasal symptoms (runny nose, blocked nose, sneezing, and itchy nose) and two ocular symptoms (gritty feeling or red or itchy eyes, and watery eyes). Each day, the patient rates the severity of each individual symptom using this scale. Results are entered in an electronic diary. The main symptom sum-score is calculated for each patient with a maximum of 18. The VAS is a general simple quantitative method that may be used for the evaluation of the severity of AR.: a 10-cm line to grade the severity of symptoms from “no symptoms” (0 cm) to “the highest level of symptoms” (10 cm) is utilized. Clinical practice guidelines have suggested a classification in which “mild” AR = 0 to 3 cm, “moderate” AR = 3.1 to 7 cm, and “severe” AR = 7.1 to 10 [14].

Statistical analyses

Statistical analyses were performed using SPSS Version 26.0 (IBM Corp., Armonk, NY, USA) for the calculation of descriptive and inferential statistics. Independent samples t-test were used to compare the groups (CPM+X vs. PLB) in continuous variables. The effects of CPM+X and PLB were evaluated using a 2 x 6 repeated-measures ANOVA (analysis of variance) with Bonferroni alfa adjustment for time effects from day 1: treatment (CPM+X vs. PLB) x time (day 1 vs. day 5 vs. day 10 vs. day 15 vs. day 20 vs. day 25 vs. day 30).

## Results

**Figure 1:**
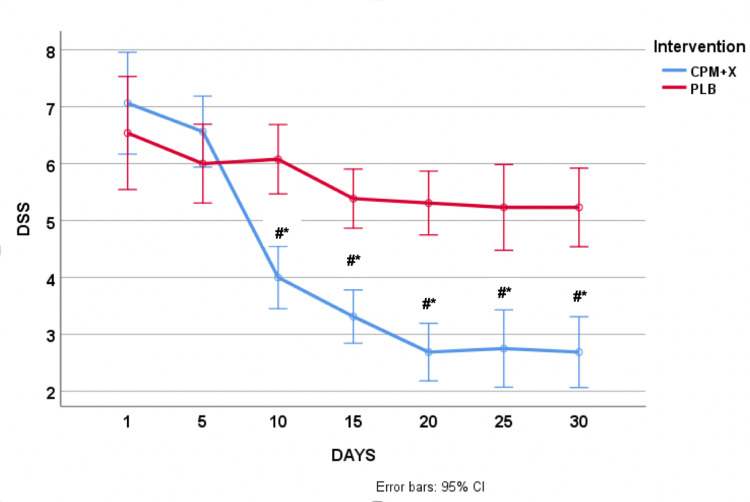
Changes in allergy DSS in response to CPM+X and placebo. Data are presented as mean ± 95% CI. #p < 0.01 vs. PLB. *p < 0.001 vs. day 1. DSS, daily symptoms score, CPM+X, chlorpheniramine maleate plus xylitol

**Figure 2:**
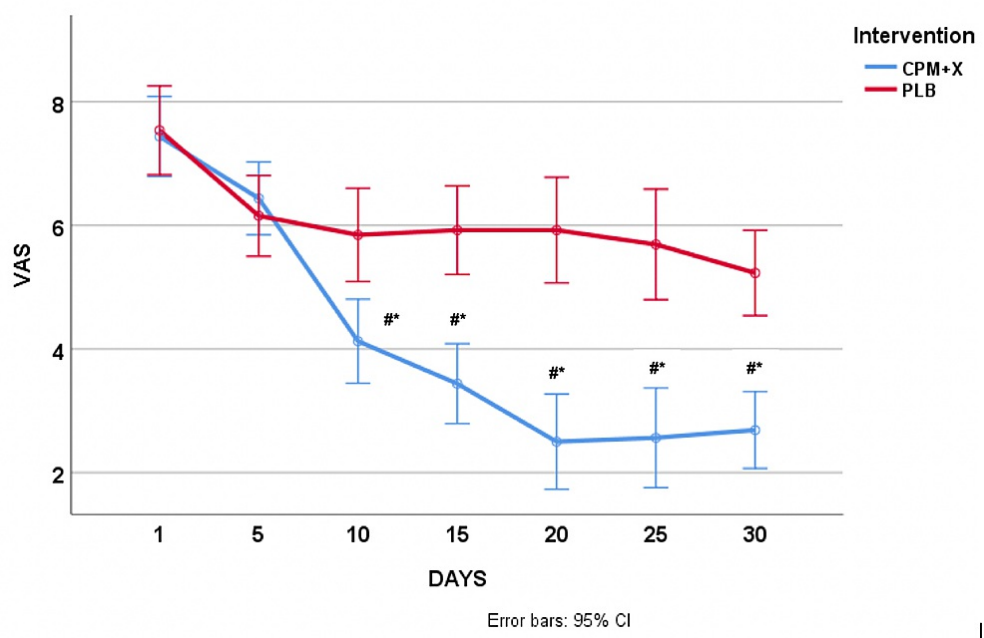
Changes in VAS in response to CPM+X and placebo. Data are presented as mean ± 95% CI. #p < 0.01 vs. PLB. * p < 0.001 vs. day 1. VAS, visual analog scale; CPM+X, chlorpheniramine maleate plus xylitol

Data are presented as M ± SME (mean ± standard error of mean). Independent samples t-test revealed no statistically significant (p > 0.05) difference between the groups (CPM+X vs. PLB) at the start of the trial in both DSS (7.1 ± 2.1 vs. 6.5 ± 1.1) and VAS (7.4 ± 1.2 vs. 7.5 ± 1.3) scores. ANOVA with repeated revealed a significant treatment x time interaction such that the CPM+X group had a significant decrease (p < 0.05) in both DSS (∆-3.0 ± 2.7) and VAS (∆-3.8 ± 2.0) scores compared to PLB after the 30 days. The difference in DSS and VAS scores between the groups was evident just after five days (day 10) of using CPM+X (Figures 1, 2). The CPM+X scores were significantly lower (p < 0.008) starting from day 10 compared with day 1, whereas there were no statistically significant (p > 0.008) changes in the PLB during the 30-day treatment window. Besides some mild discomfort felt by patients immediately after the application of the spray, there were neither adverse reactions nor side effects reported by the participants. None of the patients in the treatment group developed infectious rhinitis during the treatment.

## Discussion

This study sought to evaluate the efficacy of intranasal delivery of CPM+X for the treatment of AR. The main novel findings of the study are in agreement with our hypothesis as results demonstrate that the use of an intranasal use spray with CPM+X in an outpatient setting is an effective and safe means to improve symptoms of AR in adult patients. Intranasally delivered CPM+X could be a viable means to treat AR while avoiding some of the main side effects of other treatments for the treatment of AR.

Intranasal corticosteroids are considered the first line of treatment for AR owing, in part, to their efficacy and route of administration, but the use of H1 blockers such as CPM through the nasal route has not been fully explored [2,15]. A prior report by Van Toor et al. demonstrated that CPM may be delivered intranasally at two dose levels to obtain a systemic bioavailability similar to a single 4 mg oral dose [9]. Also, aerosolized CPM has been used, at least experimentally, for the treatment of exercise-induced asthma and bronchospasm [8]. Based on the aforementioned studies, we speculate that the systemic levels of intranasally delivered CPM in this study did not exceed 1 mg, which may explain why the patients did not report drowsiness in the treatment group without affecting the efficacy of the intervention. Interestingly, none of the participants developed infectious rhinitis, which is an important side effect associated with chronic nasal corticosteroid use [15].

Although outside the design and scope of the present pilot, there some potential underlying mechanisms explaining the effectiveness of the nasal spray with CPM+X for reducing AR symptomatology in the tested population of patients. First, the H1 blocking capabilities of CPM suppress the main mediators associated with the allergic process, deactivate eosinophilic anti-apoptotic modulators, and downregulate aquaporin activation, all of which are associated with AR symptomatology [2,6,7]. Second, CPM has been shown to display broad-spectrum antiviral characteristics, which may reduce the probabilities to acquire viral rhinitis [5]. Lastly, intranasal irrigations with xylitol have been previously shown to be effective in decreasing AR [10]. Additionally, xylitol has been shown to inhibit the growth of Streptococcus pneumoniae and other nasopharyngeal bacteria and hence may lead to beneficial microbiome-modulating effects that aid in the reduction of AR symptoms [16,17].

The study demonstrates an efficacious combination of a well-known antihistamine and xylitol in addressing AR. This is a pilot study to evaluate the initial dosing and patient’s response before pursuing a large-scale multicenter trial. Furthermore, a complete microbiome analysis to detect the impact of CPM +X on the microbiome was not conducted, which is one of the study limitations. However, the impact of the microbiome in the pathogenesis of AR should not be overlooked as it has been gaining attention among researchers in the allergy and immunology fields [4,18,19].

## Conclusions

The present data suggest that nasal CPM+X use effectively improves AR symptoms, with minimal unwanted side effects reported. The effects of the treatment are evident after five days of use without any major side effects. Studies with more patients, as well as the investigation of the long-term effects of CPM+X for the treatment of other chronic respiratory disorders such as asthma and sleep apnea as well as the potential microbiome-modulating effects, warrant further investigation.
